# Cefiderocol: early clinical experience for multi-drug resistant gram-negative infections

**DOI:** 10.1128/spectrum.03108-23

**Published:** 2024-01-11

**Authors:** Amer El Ghali, Ashlan J. Kunz Coyne, Kristen Lucas, Molly Tieman, Xhilda Xhemali, Suet-ping Lau, Gabriela Iturralde, Andrew Purdy, Dana J. Holger, Esther Garcia, Michael P. Veve, Michael J. Rybak

**Affiliations:** 1 Department of Pharmacy Practice, Eugene Applebaum College of Pharmacy and Health Sciences, Wayne State University, Detroit, Michigan, USA; 2 Department of Pharmacy, Indiana University Health, Bloomington, Indiana, USA; 3 Department of Pharmacy, Cleveland Clinic, Cleveland, Ohio, USA; 4 Department of Pharmacy, Orlando Health, Orlando, Florida, USA; 5 Department of Pharmacy, Memorial Hospital West, Pembroke Pines, Florida, USA; 6 Department of Pharmacy Practice, Barry and Judy Silverman College of Pharmacy, Nova Southeastern University, Fort Lauderdale, Florida, USA; 7 Department of Pharmacy, Henry Ford Hospital, Detroit, Michigan, USA; 8 Department of Medicine, Division of Infectious Diseases, School of Medicine, Wayne State University, Detroit, Michigan, USA; 9 Department of Pharmacy Services, Detroit Receiving Hospital, Detroit Medical Center, Detroit, Michigan, USA; Emory University School of Medicine, Atlanta, Georgia, USA

**Keywords:** Cefiderocol, *Acinetobacter baumannii*, *Pseudomonas aeruginosa*, carbapenem-resistant, gram-negative resistance

## Abstract

Multi-drug resistant gram-negative bacteria present a significant global health threat. Cefiderocol (CFDC), a siderophore cephalosporin, has shown potential in combating this threat, but with the currently available data, its role in therapy remains poorly defined. This multi-center, retrospective cohort study evaluated the real-world application of CFDC across six U.S. medical centers from January 2018 to May 2023. Patients aged ≥18 years and who had received ≥72 hours of CFDC were included. The primary outcome was a composite of clinical success: survival at 30 days, absence of symptomatic microbiologic recurrence at 30 days following CFDC treatment initiation, and resolution of signs and symptoms. Secondary outcomes included time to CFDC therapy and on-treatment non-susceptibility to CFDC. A total of 112 patients were included, with median (interquartile range [IQR]) APACHE II scores of 15 (19–18). Clinical success was observed in 68.8% of patients, with a mortality rate of 16.1% and comparable success rates across patients infected with carbapenem-resistant gram-negative infections. The most common isolated organisms were *Pseudomonas aeruginosa* (61/112, 54.5%, of which 55/61 were carbapenem-resistant) and carbapenem-resistant *Acinetobacter baumannii* (32/112, 28.6%). Median (IQR) time to CFDC therapy was 77 (14–141) hours. Two patients experienced a non-anaphylactic rash as an adverse drug reaction. On-treatment non-susceptibility to CFDC was found in six patients, notably due to *P. aeruginosa* and *A. baumannii*.

**IMPORTANCE:**

CFDC was safe and clinically effective as a monotherapy or in combination in treating a variety of carbapenem-resistant gram-negative infections. Further prospective studies are warranted to confirm these findings.

## INTRODUCTION

The escalating challenge of antimicrobial resistance stands as a formidable threat to global health. This concern is primarily fueled by gram-negative bacteria, which are becoming increasingly resistant to existing therapeutic measures (1–4). Especially alarming are carbapenem-resistant *Enterobacterales* (CRE), carbapenem-resistant *Acinetobacter baumannii* (CRAB), and carbapenem-resistant *Pseudomonas aeruginosa* (CRPA), which are often associated with nosocomial infections and have contributed to an upward trajectory in morbidity and mortality rates (5, 6). Although antimicrobial stewardship programs have assisted in addressing practice-level contributions to rising resistance, these pathogens require the continued development of novel treatments. Ironically, pharmaceutical companies are left with several challenges to research and development, leading to the production of very few novel antimicrobial agents in the last decade (7).

Cefiderocol (CFDC), a novel siderophore cephalosporin, was developed as a promising countermeasure to this growing issue. Mechanistically, CFDC leverages the iron-transport systems of gram-negative bacteria to penetrate the outer cell membrane (8). This strategic approach to combat carbapenem resistance represents a significant step forward in antimicrobial development. CFDC has demonstrated potent *in vitro* activity against a broad range of multi-drug resistant (MDR) gram-negative resistant pathogens and phenotypes, including extended-spectrum β-lactamases (ESBL) and AmpC, metallo- and serine-carbapenemases, as well as MDR pathogens that exhibit resistance mechanisms unrelated to ß-lactamases (8–13).

Following the results of the APEKS-cUTI and APEKS-NP studies, the Food & Drug Administration (FDA) approved CFDC for the treatment of complicated urinary tract infections, hospital-acquired bacterial pneumonia (HABP), and ventilator-associated bacterial pneumonia (VABP) (14, 15). CFDC was also tested in the phase III CREDIBLE-CR trial, which focused on patients with carbapenem-resistant infections (16). However, based on the results of this trial that demonstrated an increase in all-cause mortality with CFDC compared to the best available therapy, the FDA label includes a warning regarding this potential risk, an observation primarily associated with *A. baumannii* infections, particularly in cases of pneumonia and bloodstream infections or sepsis. As such, these results have cast some uncertainty among clinicians regarding the place of therapy of CFDC.

In this study, we aim to share clinical experiences with CFDC across a variety of medical centers, providing a valuable opportunity to evaluate the real-world application and clinical success of CFDC in tackling carbapenem-resistant infections.

## MATERIALS AND METHODS

This study was a multi-center, retrospective, cohort study, including six distinct academic medical centers across the United States, where CFDC was initiated from January 2018 to May 2023. Approval was obtained from each participating center’s Institutional Review Board with a waiver for informed consent. Patients were included if they were ≥18 years old and were on CFDC for ≥72 hours, with all subsequent courses requiring a gap of ≥90 days. The primary outcome was a composite of clinical success, defined as survival, the absence of symptomatic microbiologic recurrence (isolation of the same bacterial species following ≥7 days of CFDC treatment) within 30 days following CFDC treatment initiation, and resolution of signs and symptoms (17–19). Secondary outcomes included the individual components of the composite clinical success outcome and non-susceptibility to CFDC defined by the FDA (20). All susceptibility data were extracted from the medical record using site-specific interpretive criteria, which varied according to the laboratory. Additionally, clinically relevant subgroups such as those with certain bacterial species and those administered CFDC as a monotherapy or as a combination regimen were examined.

Manual retrospective chart abstraction was used by co-investigators, and all cases were audited by two or more investigators. Any missing data or discrepancies were corrected before data analysis. Hospital-acquired infections were defined as those having positive cultures ≥ 48 hours after hospital admission. Time to CFDC therapy, in hours, was defined from index culture collection to the first administration of an CFDC for ≥48 hours, which was considered susceptible by FDA breakpoints (21–24). HABP and VABP were defined by IDSA criteria (25). Surgical procedures were extracted from the admission for which they received CFDC and included procedures such as intravenous catheter removal, valvular repair, valvular replacement, invasive device removal, incision and drainage, debridement, and amputation. Combination therapy was defined as receiving any concomitant antibiotic(s) with CFDC for ≥48 hours for CFDC-targeted infections. Carbapenem resistance was defined as testing resistant to ≥1 carbapenem antibiotic (26, 27). Similarly, CRE was defined by the CDC’s criteria of testing resistant to at least one carbapenem antibiotic or producing a carbapenemase enzyme. Minimum inhibitory concentration (MIC) breakpoints were determined by the FDA interpretive criteria. The severity of illness was assessed using the following three indices: the age-unadjusted Charlson Comorbidity Index (CCI), the Acute Physiology and Chronic Health Evaluation Score (APACHE II), and the Sequential Organ Failure Assessment (SOFA) score (21, 28, 29). For the APACHE-II and SOFA scores, the most extreme values recorded within the first 24 hours of receiving CFDC were used for eligible patients. The CCI was determined based on the reported ICD-10 codes and/or past medical history as noted by physicians.

The Chi-squared or Fisher exact test was used for comparison with categorical variables, whereas the *t*-test and Mann–Whitney U test were used to compare nominal and continuous variables, respectively. IBM SPSS software, version 29.0.1.0 (SPSS, Inc., Chicago, IL, USA), was used for all statistical analyses.

## RESULTS

### Patient characteristics

A total of 112 unique patients were included in the study (Table 1). All hospitals involved in the study were academic medical centers, and the distribution of cases was fairly equal (median: 25, range: 10–29). The median interquartile range (IQR) age of the patients was 58 (44–67) years, with 64.3% (72/112) being male. Most patients were Caucasian (64/112, 57.1%), followed by African American (30/112, 26.8%), and Latino (14/112, 12.5%). The median Body Mass Index (BMI) at baseline was 25.3 kg/m² (IQR, 21–32). Of these, 28.6% (32/112) were classified as obese with a BMI of ≥30 kg/m², and 8.9% (10/112) were underweight, with a BMI <18.5 kg/m². In terms of admission location, 43.8% (49/112) were admitted from home and 25.0% (28/112) were transferred from an outside hospital. The most common underlying comorbidity was diabetes (37.5%) followed by moderate to severe chronic kidney disease (CKD) (22.3%) and immunocompromised (22.3%).

**TABLE 1:** Patient baseline characteristics^*a*^

Characteristics	(*n* = 112)
Age, years, median (IQR)	58 (44–67)
Age ≥ 65 years	41 (36.6)
Male	72 (64.3)
BMI, kg/m², median (IQR)	25.3 (21–32)
Obese (BMI, ≥30 kg/m^2^)	32 (28.6)
Underweight (BMI, <18.5 kg/m^2^)	10 (8.9)
Race	
Caucasian	64 (57.1)
African American	30 (26.8)
Latino	14 (12.5)
Admission Location	—
Home	49 (43.8)
Transfer from outside hospital	28 (25.0)
Nursing home, skilled nursing facility, long-term care facility	20 (17.9)
Inpatient rehabilitation facility	7 (6.3)
Long-term acute care facility	6 (5.4)
Other	2 (1.8)
Comorbid Conditions	
Diabetes	42 (37.5)
Moderate to severe CKD	25 (22.3)
Immunocompromised	25 (22.3)
COPD	22 (19.6)
Cerebrovascular disease (stroke, TIA)	21 (18.8)
Peripheral vascular disease (DVT, chronic venous stasis)	20 (17.9)
Heart failure	16 (14.3)
Cancer	14 (12.5)
MDR Risk Factors	
Antimicrobials ≥24 hours in the past 90 days	81 (72.3)
Hospitalization ≥48 hours in the past 90 days	78 (69.6)
Prior infection with a resistant organism	54 (48.2)
Colonization with resistant organisms	38 (33.9)
Admitted from a nursing home or extended care facility	31 (27.7)
Surgery in the past 30 days	23 (20.5)
Home wound care	19 (17.0)
ICU Admission	74 (66.1)
One admission	56 (75.7)
2–3 admissions	18 (24.3)
Index culture collected in ICU	48 (42.9)
APACHE II, median (IQR)	15 (9–18)
≥20	24 (21.4)
Charlson Comorbidity Index, median (IQR)	4 (3-7)
SOFA score, median (IQR)	5 (3–7.75)

^*a*^ Data reported as *n* (%) unless otherwise specified; IQR, interquartile range; BMI, body mass index; CKD, chronic kidney disease; COPD, chronic obstructive pulmonary disease; MDR, multi-drug resistant; TIA, trans-ischemic attack; DVT, deep vein thrombosis. ICU admission refers to the hospitalization period when a patient receives cefiderocol. MDR risk factors are based on the health care-associated pneumonia from 2005 American Thoracic Society Infectious Diseases Society of America [ATS IDSA] guidelines (30).

The median (IQR) APACHE II, CCI score, and SOFA score were 15 (9–18), 4 (3–7), and 5 (3–7.75), respectively. Among the studied patients, 72.3% (81/112) had been exposed to antimicrobials for at least 24 hours in the prior 90 days. This subset comprises 28.6% (32/112) who received meropenem, 8.0% (9/112) treated with ceftazidime-avibactam, 3.6% (4/112) given ceftolozane/tazobactam, and 1% (1/112) administered meropenem-vaborbactam. Furthermore, 69.6% (78/112) had a hospital stay of a minimum of 48 hours within the same timeframe. Notably, 66.1% (74/112) of these patients had at least one ICU admission during hospitalization, with 42.9% (48/112) having their index culture collected during their ICU stay.

### Infection characteristics

A complete list of infection characteristics is summarized in Table 2. Most infections were hospital-acquired, 68.8% (77/112), while the time from admission to culture collection varied widely, with a median (IQR) of 159 (22–722) hours. The most common infection source was the lower respiratory tract, 50.9% (57/112). Among these, VABP occurred in 31.3% (35/112) of patients and HABP occurred in 14.3% (16/112). Skin and soft tissue infections (SSTI) were the next most frequent infection types seen in 18.8% (21/112) of patients. Other infection sources included 10.7% (12/112) intra-abdominal, 6.3% (7/112) urinary tract, 4.5% (5/112) osteomyelitis, and 4.5% (5/112) from unknown sources.

**TABLE 2:** Infection characteristics^*a*^

Characteristics	(*n* = 112)
Hospital-acquired infection	77 (68.8)
Time to culture collection, hours, median (IQR)	159 (22–722)
Infection Source	
Nosocomial Pneumonia	57 (50.9)
Ventilator-associated bacterial pneumonia	35 (31.3)
Hospital-acquired bacterial pneumonia	16 (14.3)
Skin and soft tissue	21 (18.8)
Intra-abdominal	12 (10.7)
Urinary tract	7 (6.3)
Osteomyelitis	5 (4.5)
Unknown	5 (4.5)
Positive blood cultures	25 (22.3)
Isolated Organisms^*b*^	
*Pseudomonas aeruginosa*	61 (54.5)
Carbapenem-resistant	55/61 (90.2)
*Acinetobacter baumannii*	32 (28.6)
Carbapenem-resistant (CRAB)	32/32 (100)
*Klebsiella pneumoniae*	15 (13.4)
Carbapenem-resistant	8/15 (53.3)
*Stenotrophomonas maltophilia*	7 (6.3)
Carbapenem-resistant	7/7 (100)
*Escherichia coli*	6 (5.4)
Carbapenem-resistant	6/6 (100)
*Achromobacter xylosoxidans*	4 (3.6)
Carbapenem-resistant	4/4 (100)
Gram-positive isolate	14 (12.5)
Carbapenem-resistant gram-negative infection	110 (98.2)
Carbapenem-resistant *Enterobacterales* (CRE)	28 (25.0)
Polymicrobial infection	51 (45.5)

^*a*^ Data reported as *n* (%) unless otherwise specified.

^*b*^ Two patients had two carbapenem-resistant isolates.

In terms of isolated organisms, *P. aeruginosa* was the most common, isolated in 54.5% (61/112) of patients, with carbapenem resistance seen in 90.2% (55/61) of these isolates. Other organisms isolated included *A. baumannii* (28.6% (32/112), all of which were carbapenem-resistant) and *Klebsiella pneumoniae* (13.4% (15/112), with carbapenem resistance seen in 53.3% (8/15) of these cases). Blood cultures were positive in 22.3% (25/112) of patients.

Almost all patients had a carbapenem-resistant gram-negative infection (98.2%, 110/112), with CRE present in 25.0% (28/112). Metallo-ß-lactamases (MBL) were found in 52.6% (10/19) of patients who underwent testing, of those nine were classified as New Delhi metallo-ß-lactamase (NDM) and one was classified as Verona Integron metallo-ß-lactamase (VIM). Polymicrobial infections, involving multiple types of bacteria, were observed in 45.5% (51/112) of patients and 68.8% (22/32) of *A. baumannii* infections were polymicrobial.

**Fig 1:**
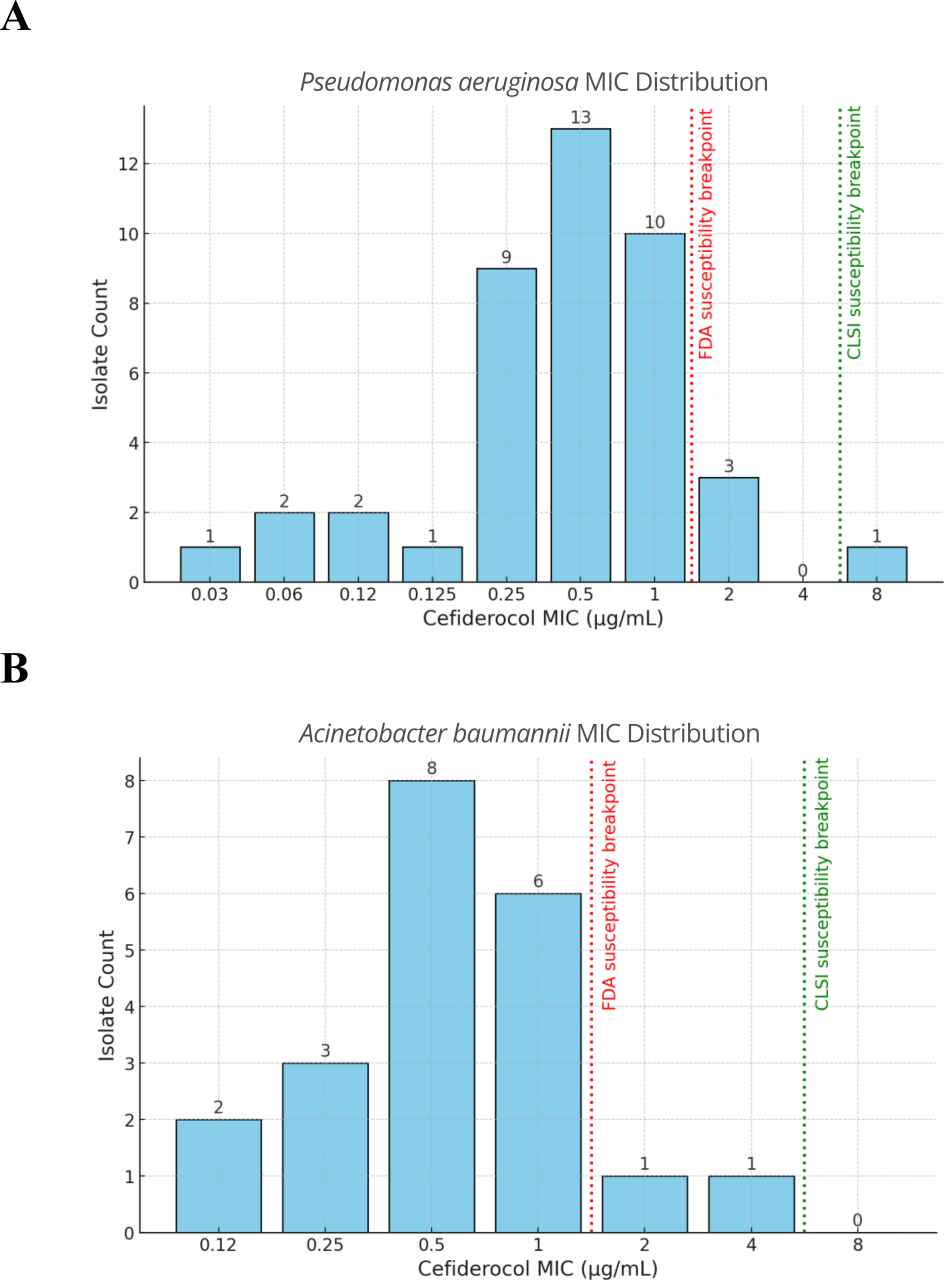
Minimum inhibitory concentration (MIC) distribution of *Pseudomonas aeruginosa* and *Acinetobacter baumannii*. (A) Cefiderocol MIC distribution of multi-drug and carbapenem-resistant *P. aeruginosa* isolates, *n* = 43. (B) Cefiderocol MIC distribution of carbapenem-resistant *A. baumannii*, *n* = 21; FDA, Food and Drug Administration; CLSI, Clinical and Laboratory Standards Institute.

In terms of consultations and treatments, most patients had an infectious disease consult (97.3%, 109/112) and 35.7% (40/112) had a surgical intervention. Active antibiotics were administered before the initiation of CFDC in 24.1% (27/112) of patients (Table 3). The median (IQR) time to CFDC was 77 (14–141) hours. CFDC was administered within 48 hours of index culture in 37.1% (42/112) of patients, and the median (IQR) CFDC duration was 9 (6–13) days. IV combination therapy with CFDC was used in 34.8% (39/112) of patients, with IV colistin-polymyxin (7.1%, 8/112), ceftazidime-avibactam (6.3%, 7/112), and aminoglycosides (5.4%, 6/112) being the most concomitantly administered antibiotics. The susceptibility of isolated pathogens to various antibiotics was also assessed (Fig. 1). For instance, *A. baumannii* (*n* = 21) and *P. aeruginosa* (*n* = 42) had MIC_50_ and MIC_90_ values of 0.5 and 1.0 µg/mL, respectively. Interestingly, CFDC non-susceptibility developed in 2/21 and 4/42 of cases with *A. baumannii* and *P. aeruginosa,* respectively.

**TABLE 3:** Treatment characteristics^*a*^

Characteristics	(*n* = 112)
Length of Stay, median, days	32 (16–53)
Infectious disease consult	109 (97.3)
Surgical intervention	40 (35.7)
Complications post-surgical interventions	15 (13.4)
Return to OR for repeat procedure	11 (9.8)
Transferred to ICU	2 (1.8)
Transferred to general floor	2 (1.8)
Active antibiotics before CFDC	27 (24.1)
Time to CFDC, hours, median (IQR)	77 (14–141)
CFDC within 48 hours	42 (37.1)
CFDC duration, days	9 (6-13)
IV combination therapy with CFDC	39 (34.8)
Colistin–polymyxin	8 (7.1)
Ceftazidime-avibactam	7 (6.3)
Aminoglycoside	6 (5.4)
Eravacycline	5 (4.5)
Imipenem/cilistatin/relebactam	5 (4.5)
Cefepime	4 (3.6)
Ampicillin/sulbactam	4 (3.6)
Concomitantly administered inhaled antibiotic therapy	23/57 (40.4)
On-treatment CFDC, non-susceptibility	6/35

^*a*^ Data reported as *n* (%) unless otherwise specified; OR, operating room; ICU, intensive care unit; CFDC, cefiderocol; IV, intravenous.

### Outcomes

**Fig 2:**
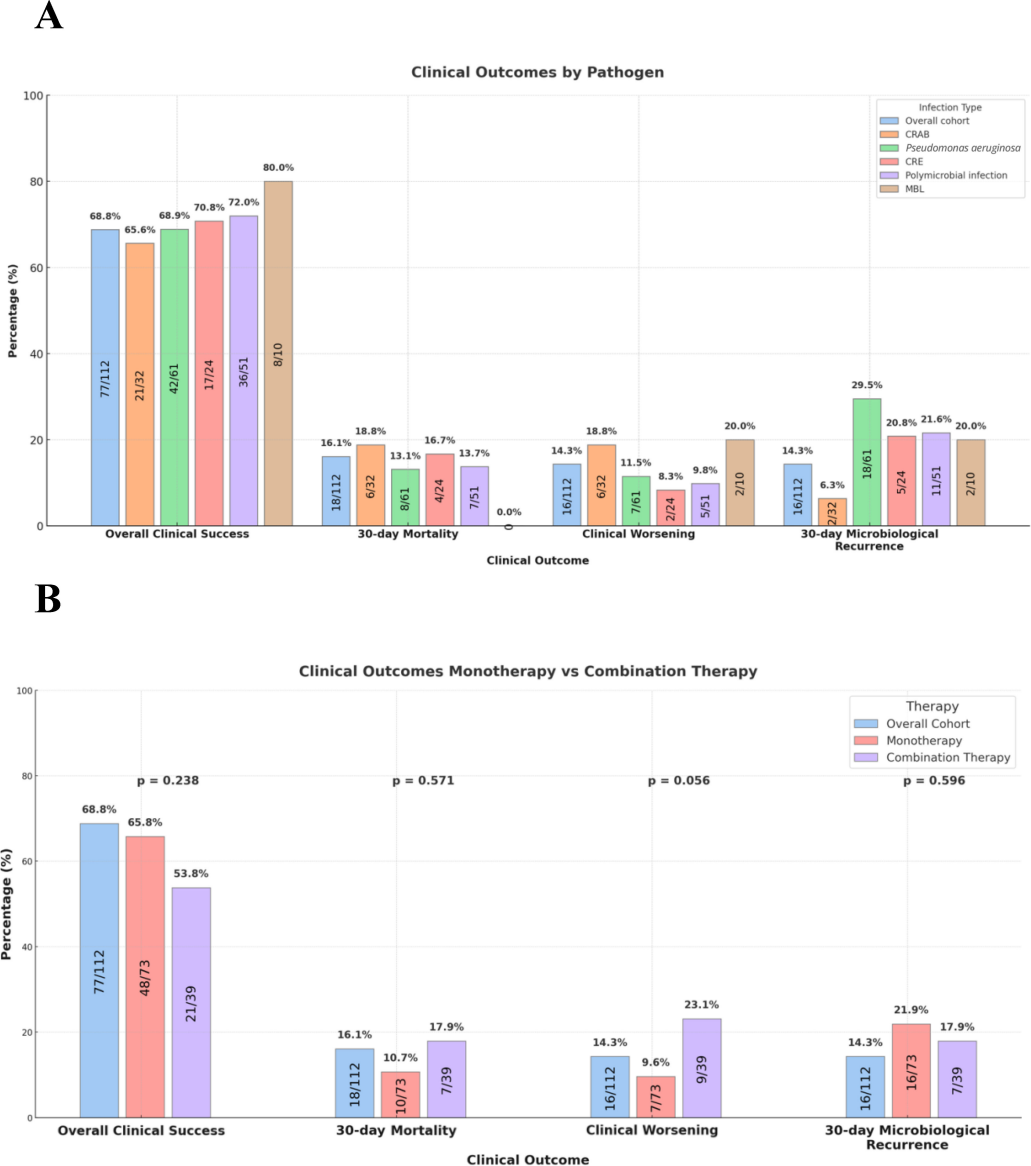
Outcomes by subgroups of Interest. (A) Clinical outcomes comparing the entire cohort to pathogen-specific sub-groups of interest; CRAB, carbapenem-resistant *Acinetobacter baumannii*; CRE, carbapenem-resistant *Enterobacterales*; MBI, metallo-B-lactamase. (B) Clinical outcomes comparing patients who received combination therapy (*n =* 39) versus monotherapy (*n =* 73). Associated *P*-values ≤ 0.05 were considered significant.

The primary outcome of clinical success was observed in 68.8% (77/112) of patients, with 30-day mortality, clinical worsening, and 30-day microbiologic recurrence occurring in 16.1% (18/112), 14.3% (16/121), and 14.3% (16/121) of patients, respectively (Fig. 2A). When examining composite clinical success and mortality by organism, similar rates were observed. Specifically, clinical success rates between patients infected with CRAB, MDR-PSA, and CRE were 65.6% (21/32), 68.9% (42/61), and 70.8% (17/24), respectively. Similarly, 30-day all-cause mortality rates were 18.8% (6/32), 13.1% (8/61), and 16.7% (4/24), respectively. Patients with polymicrobial infections also demonstrated clinical success rates of 72% (36/51), and of the 6 patients with co-occurring *A. baumannii* and *P. aeruginosa* infections, 5/6 met the clinical success endpoint. When examining the 10 patients with an MBL infection, 8/10 (7 NDM and 1 VIM) met the clinical success endpoint and 1 had *K. pneumoniae* harboring both NDM and OXA-48 carbapenemases, which was successfully treated with CFDC. Six patients went on to develop on-treatment CFDC resistance, with four because of *P. aeruginosa*, one to *A. baumannii*, and one to *K. pneumoniae*.

### Combination therapy versus monotherapy

Additionally, when comparing CFDC monotherapy to CFDC combination therapy, clinical success was observed in 65.8% (48/73) versus 53.8% (21/39) (P = 0.238) of patients, respectively (Fig. 2B). Combination therapy in these patients consisted predominantly of IV colistin/polymixin B (8/112, 7.1%), ceftazidime-avibactam (7/112, 6.3%), and aminoglycosides (6/112, 5.4%). Bivariate analysis between the two groups revealed no discernable difference in patient baseline characteristics or treatment characteristics other than receipt of combination therapy. The median (IQR) duration of antibiotic overlap was 5.02 (2.31–8.31) days. When specifically evaluating patients with *A. baumannii* infections, 15/32 received combination therapy mostly consisting of eravacycline (5/15, 33.3%), ampicillin/sulbactam (4/15, 26.7%) and IV colistin/polymixin B (4/15, 26.7%).

### Safety

Only two patients experienced an adverse drug reaction (ADR) while receiving CFDC, which in both cases was a non-anaphylactic rash. Both patients had a documented history of allergic reactions to penicillin, which had previously resulted in itching, with or without hives. Conversely, 17 other patients with a documented history of allergic reactions to ß-lactams varying from itching and hives to angioedema and anaphylaxis experienced no allergic reactions while taking CFDC.

## DISCUSSION

This study presents one of the largest published data sets on the real-world experiences with CFDC in the treatment of predominantly carbapenem-resistant gram-negative infections. These experiences provide insight into clinical scenarios, treatment characteristics, health outcomes, and safety concerns associated with CFDC that may be encountered in the real-world clinical setting, further clarifying the utility of CFDC. Our findings suggest that CFDC could be a promising treatment option for a range of carbapenem-resistant gram-negative infections, as evidenced by the observed clinical success and mortality rates of 68.8% and 16.1%, respectively.

Importantly, clinical success rates did not demonstrate significant fluctuations based on the organism, resistance phenotype, or whether CFDC was used in combination or as a monotherapy. One contentious finding of the CREDIBLE-CR study was the higher observed mortality rates in patients with CRAB infections compared to those receiving the best available therapy, a result that prompted warnings from the FDA (16). In contrast to the CREDIBLE-CR trial, which reported 28-day mortality rates of 38% (50% at the end of the study) for CRAB, our study demonstrated a significantly lower rate of 18.8% at 30 days (16). When comparing patient populations, several similarities were noted, including median BMI, APACHE II scores, CCI, and the proportion of VABP, HABP, and bloodstream infections. Despite these similarities, the variance in mortality rates between our study and the CREDIBLE-CR trial could be attributed to differences in age, proportion of APACHE II scores ≥ 20, and clinical management strategies. However, in comparison to the CREDIBLE-CR trial, mortality rates due to CR-*P. aeruginosa* and CRE, were not different. We also observed high degrees of clinical success (80%) against confirmed MBL pathogens that included 9 NDM- and 1 VIM-producing *P. aeruginosa* isolate, which confirmed the 70.8% clinical cure rates observed in a subgroup of MBL-harboring pathogens from the APEKS-NP and CREDIBLE-CR studies (15, 16). With very few options for MBL-producing pathogens, CFDC remains an important antimicrobial and has been recommended along with a combination of aztreonam and avibactam (5, 31, 32). Another important finding from our study was that there was no difference in clinical outcomes between patients who received CFDC combination therapy or monotherapy, which generally supports other findings that have found no difference (33).

Another area that may be of concern is the emergence of resistance to CFDC during therapy. Although non-susceptibility to CFDC through ongoing surveillance data remains low, there are increasing reports of the emergence of on-treatment CFDC resistance (15, 16, 33–36). In our analysis, we found six patients who developed resistance to CFDC during treatment based on CLSI interpretive criteria, signaling potential adaptive responses within these bacterial populations in the presence of CFDC. Additionally, four patients were found to harbor CFDC-resistant bacteria at baseline, which suggests the possibility of nosocomial acquisition of isolates previously exposed to CFDC. This trend aligns with a systematic review, which reported an increasing number of cases of *in vivo* emerging CFDC resistance, particularly involving co-expression of multiple β-lactamases in combination with permeability defects as the primary resistance mechanism having never been exposed previously (37). Furthermore, a study focused on carbapenem-resistant CRE found that 25% of the isolates exhibited non-wild-type CFDC resistance, suggesting a combination of heterogeneous mechanisms behind CFDC resistance, with no single consistent antimicrobial resistance marker identified (38). These findings underscore the complexity of CFDC resistance and the necessity for continued surveillance and research to elucidate the underlying mechanisms. Furthermore, they highlight the importance of judicious CFDC use in clinical settings to mitigate the risk of resistance development and spread. Future investigations should focus on understanding the molecular basis of CFDC resistance, as well as developing strategies to counteract the emergence of resistant strains.

Although several papers have documented the real-world outcomes of CFDC, the majority of these are single-center studies with fewer than 50 total patients or pathogen-specific groups (33, 39). Moreover, many of these studies lack number and diversity of carbapenem-resistant organisms*,* which is a strength of our study (33, 36, 39, 40). The most extensive study to date was a recently published retrospective cohort consisting of 48 patients from the VA medical system. Interestingly, the primary pathogen in this study, like our own, was *P. aeruginosa*. Clinical failure and 30-day all-cause mortality was observed in 35.4% (17/48) and 27.1% (13/48) of patients, respectively. However, the lack of APACHE II and SOFA scores in the report makes it challenging to gauge and compare the disease severity across studies. In contrast to our study population, the patients in the VA cohort were considerably older with a median (IQR) age of 70.5 (60.5–74) years, 41.7% (20/48) had malignancies, and 47.9% (23/48) were dealing with complicated diabetes. Further complicating the analysis, specific CFDC MICs for the pathogens were not provided. Instead, the results were interpreted by the in-house microbiology lab based on varying breakpoints set by the FDA, EUCAST, and CLSI. This lack of standardized reporting makes direct comparisons and aggregate analysis more challenging. Additionally, we observed a higher incidence of SSTI among our patients (19%, 21/112). This could be attributed to the prevalent comorbid conditions with immunosuppressive properties, such as diabetes and malignancy that were experienced in this study. Notably, out of the 21 patients with SSTI, five had positive blood cultures and nine were admitted to the ICU during their CFDC treatment. However, previous studies describing CFDC use have reported a similar incidence of SSTI, especially when treating *P. aeruginosa* (39, 41).

There are several limitations to this study. First, this was a retrospective study, which may introduce selection bias. Despite designing it to be multicenter for diversity, site-specific variance in treatment protocol could still potentially affect interpretation. Second, we did not have an active comparator, which may limit generalizability. Third, the absence of CFDC MIC data for a substantial number of patients and the variation in antimicrobial susceptibility testing (AST) and interpretive criteria unique to each study site. These omissions are consequential as MIC data is key for assessing the efficacy of CFDC and gauging the resistance level of the pathogen. Particularly for these MDR pathogens, which can exhibit diverse resistance mechanisms, missing MIC data can result in an underestimation of resistance within our study population. However, we do believe this is an accurate representation of current AST interpretation by many microbiology labs across the United States. Furthermore, without this information, we are unable to correlate clinical outcomes with the degree of CFDC resistance, a critical factor for future patient management and treatment planning. Fourth, although the median demographic characteristics in our study might appear similar to other studies, the distribution within certain variables, such as APACHE II scores and age, may differ. Finally, we were unable to assess whether a patient had adequate source control other than whether or not they had a surgical procedure, which may affect the outcomes of patients with skin and soft tissue or intraabdominal infections.

Despite these limitations, the culmination of experiences provided in this study offers clinicians the largest set of real-world data that has currently been published, providing clinical insights into combination therapy versus monotherapy, CFDC resistance, and a diverse assortment of carbapenem-resistant gram-negative pathogens. We found that these experiences add to the body of evidence that supports the use of CFDC against MDR gram-negative pathogens, particularly CRAB, CRE, and CRPA. With this evidence, we do believe further prospective and comparative data investigating the use of CFDC in CRAB-associated bloodstream and pulmonary infections are crucial to further defining CFDCs’ role in therapy as well as translational studies to investigate the relationships between clinical pathogens and the emergence of CFDC resistance.
